# Clinical Practice-Based Failure Modes and Root Cause Analysis of Cone Beam CT-Guided Online Adaptive Radiotherapy of the Pelvis

**DOI:** 10.3390/cancers17091462

**Published:** 2025-04-26

**Authors:** Dandan Zheng, Michael Cummings, Hong Zhang, Alexander Podgorsak, Fiona Li, Olga Dona Lemus, Matthew Webster, Neil Joyce, Erika Hagenbach, Kevin Bylund, Haoming Qiu, Matthew Pacella, Yuhchyau Chen, Sean Tanny

**Affiliations:** 1 Department of Radiation Oncology, University of Rochester, Rochester, NY 14627, USA; michael_cummings@urmc.rochester.edu (M.C.); hong_zhang@urmc.rochester.edu (H.Z.); alexander_podgorsak@urmc.rochester.edu (A.P.); fenglifiona_li@urmc.rochester.edu (F.L.); matt_webster@urmc.rochester.edu (M.W.); neil_joyce@urmc.rochester.edu (N.J.); erika_hagenbach@urmc.rochester.edu (E.H.); kevin_bylund@urmc.rochester.edu (K.B.); haoming_qiu@urmc.rochester.edu (H.Q.); matthew_pacella@urmc.rochester.edu (M.P.); yuhchyau_chen@urmc.rochester.edu (Y.C.); sean_tanny@urmc.rochester.edu (S.T.); 2 Department of Radiation Oncology, University of Miami, Coral Gables, FL 33146, USA; oxd407@med.miami.edu

**Keywords:** adaptive radiotherapy, failure modes, root cause analysis, CBCT, Ethos, prostate cancer, pelvis

## Abstract

**Simple Summary:**

Cone beam computed tomography (CBCT)-guided online adaptive radiotherapy (oART) has introduced a paradigm shift in radiotherapy by enabling online plan adaptation based on daily anatomical changes. While its automation enables and streamlines workflow, our retrospective analysis of over 1000 pelvic oART sessions identified a range of failure modes (FMs) related to system-driven constraints, patient-specific anatomical variations, and treatment execution challenges. These included contouring mismatches, software-driven irregularities, intrafractional anatomy changes, and limitations in fallback plans. The system’s automated workflow reduces human variability but also introduces rigid constraints that, in certain cases, impact treatment accuracy. Addressing these challenges requires a multidisciplinary approach, continuous quality assurance, and ongoing refinements to automation protocols. This study provides a comprehensive clinical practice-based evaluation of the challenges associated with CBCT-guided oART and highlights key areas for future improvements in adaptive radiotherapy systems.

**Abstract:**

**Background/Objectives:** Cone-beam computed tomography (CBCT)-guided online adaptive radiotherapy (oART) represents a significant advancement in radiation oncology, enabling on-couch plan adaptation to account for daily anatomical changes. While this automation improves precision and workflow efficiency, it also introduces new failure modes (FMs) and workflow irregularities. This study aimed to systematically evaluate the clinical and technical challenges associated with CBCT-guided oART implementation. **Methods:** We retrospectively analyzed over 1000 CBCT-guided oART sessions for pelvic malignancies performed at our institution. A multidisciplinary team conducted a comprehensive review to identify and classify FMs, followed by root cause analysis (RCA) to evaluate their impact on treatment safety, efficacy, and workflow robustness. **Results:** In addition to session-terminating FMs, we identified recurring failure modes across three major domains: (1) system-driven issues, such as rigid target localization and software-driven irregularities; (2) patient-driven challenges, including interfractional and intrafractional anatomical variations; and (3) treatment planning and execution failures, including excessive dose hotspots from field-of-view limitations. The system’s closed-loop automation, while streamlining processes, introduced rigid constraints in plan adaptation and fallback plan execution, occasionally leading to unintended dose discrepancies. **Conclusions:** This study provides a comprehensive clinical practice-based evaluation of CBCT-guided oART, highlighting system-specific failure modes and their implications. Addressing these challenges requires structured quality assurance processes, multidisciplinary collaboration, and continuous workflow refinement. Our findings contribute to the development of safer and more robust adaptive radiotherapy platforms and clinical workflows.

## 1. Introduction

Online adaptive radiotherapy (oART) is a significant advancement in radiation oncology, enhancing precision, accuracy, and efficacy in treatment delivery [1]. Conventional radiotherapy is based on the concept of adding margins around targets and organs at risk (OARs) to account for setup and motion uncertainties. This approach assumes patient anatomy is relatively rigid, which may not always hold true.

Modern radiotherapy technologies, such as intensity-modulated radiotherapy (IMRT) and image-guided radiotherapy (IGRT), have significantly improved target and delivery precision [2]. These advancements have enabled margin reductions and dose escalations. However, substantial deformations of both targets and OARs can occur during treatment due to tumor shrinkage or growth, variation in organ filling, organ relocation, changes in habitus, or patient weight fluctuations [3,4,5,6].

Daily oART introduces a paradigm shift by adapting and reoptimizing treatment plans in real time to account for anatomical changes, ensuring accurate dose delivery for every session. Advances in integrated hardware and software systems have made oART feasible, streamlining adaptive radiotherapy [1]. These systems streamline adaptive radiotherapy by incorporating image acquisition, contour generation, treatment planning, quality assurance (QA), and delivery. Following earlier systems based on magnetic resonance imaging (MRI), Varian Ethos (Varian Medical Systems, Palo Alto, CA, USA), a cone beam computed tomography (CBCT)-guided system, was introduced to clinical practice in 2020 with FDA approval [7,8,9]. This CBCT-based online ART system offers advantages such as fast imaging speed, high patient throughput, lower cost, and wider accessibility compared to other specialized platforms. Since its advent, Ethos has become an increasingly popular online ART platform adopted by many clinics [10,11,12,13,14,15,16,17,18,19].

Much literature has been published on the implementation of the system and its applications to treat various cancers [10,11,12,13,14,15,16,17,18,19]. Technical and clinical discussions involving the technology are growing rapidly as experience with the system continues to accumulate across institutions. As one of the early adopters, our department began oART treatments using Ethos in July 2021, performing over 1000 oART sessions on 50 adaptive pelvic patients by the time of writing.

As oART systems and workflows are new, they introduce unique steps and redefine clinical team roles. Continuous monitoring and QA, such as through failure mode and effects analysis (FMEA), are critical for ensuring safe treatment execution and identifying unforeseen failure modes or weak links [20]. The feasibility of oART relies heavily on automation, advanced algorithms, and artificial intelligence, which enable real-time adaptation but also introduce potential risks requiring careful oversight.

Despite the growing adoption of CBCT-guided oART, there is limited literature on its failure modes, highlighting the need for comprehensive studies to ensure safe and effective implementation [21,22,23,24,25]. Previous studies have often relied on emulated workflows or prospective analyses, leaving critical gaps in understanding the practical challenges of implementing oART. Our study, based on real-world practice, aims to address this gap by identifying common and novel failure modes (FMs) and irregularities encountered in over 1000 adaptive sessions for pelvic patients and offering insights for continuous quality improvement.

## 2. Materials and Methods

### 2.1. Overview of the Reviewed Pelvic oART Cases and Our Adaptive Workflow

Our clinic began implementing CBCT-guided oART on Ethos in July 2021, treating over 1000 fractions across 50 pelvic patients by January 2025. Most cases involved post-prostatectomy radiotherapy, with others including intact prostate, pelvic lymph nodes, and rectal and bladder cancers. Treatments used Varian Ethos versions 1.0 (July–October 2021) and 1.1 (since October 2021), with an upgrade to the HyperSight imaging system in April 2024.

**Figure 1:**
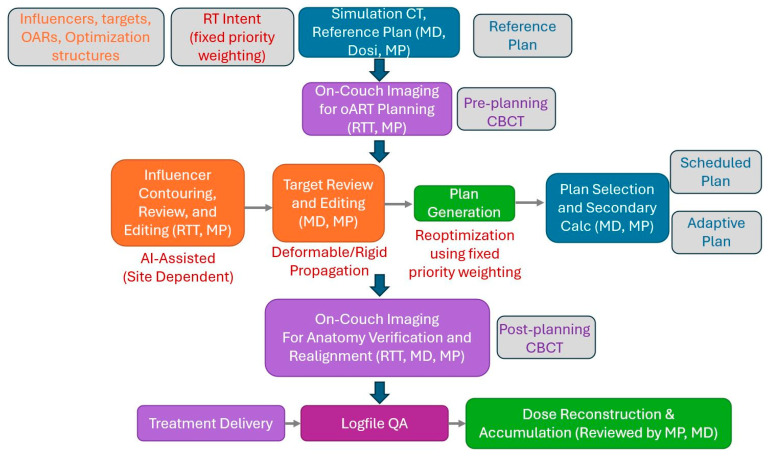
Our CBCT-oART typical workflow. Personnel involved in various steps: MD = radiation oncologist; Dosi = medical dosimetrist; MP = medical physicist; RTT = radiation therapist.

For our adaptive workflow, the attending radiation oncologist, an adaptive medical physicist, and radiation therapists were typically present during on-couch adaptations. In these pelvic adaptive cases, influencer structures such as the bladder, rectum, bowel (when applicable), and prostate (when applicable) were utilized to optimize image registration, target propagation, and treatment planning. Initially generated via AI models, these influencer structures are critical in guiding the adaptation process within the Ethos system, which employs synthetic CT (sCT) generation and automated steps to streamline segmentation, registration, and planning adjustments. Influencer structures play a key role in driving deformable image registration in synthetic CT generation, ensuring accurate mapping of daily anatomy to targets and OARs. The Ethos system incorporates fixed priority weighting based on RT intent, assigning predefined weights to clinical goals instead of a planner’s iterative tuning to guide automated decision-making in adaptive planning. A single medical dosimetrist was responsible for planning all cases. Figure 1 shows a schematic of our oART workflow steps and responsible parties.

### 2.2. Multidisciplinary Approach and Retrospective Review Methodology

To support and refine the oART process, a multidisciplinary team comprising adaptive medical physicists, radiation oncologists, medical dosimetrists, and radiation therapists met weekly. These meetings focused on systematically reviewing adaptive treatment plans, session data, and irregularities encountered during the workflow. The team also conducted ad hoc risk assessments and root cause analyses for identified irregularities.

As part of this effort, an in-depth retrospective review was conducted on over 1000 oART sessions for 50 pelvic treatment patients. The review covered all stages of the oART workflow: from reference treatment planning, session imaging, contouring, treatment planning, plan selection, and QA to treatment delivery. Primary and unique failure modes or irregularities identified during this review were analyzed through root cause assessments with input from the multidisciplinary team. This process also included evaluating the potential clinical impacts of these FMs and documenting strategies for risk mitigation and workflow improvement. Findings from this review informed iterative updates to the oART process, enhancing workflow safety and treatment quality.

Due to their critical impact on treatment continuity, aborted sessions were presented separately from other FMs. These events, while relatively infrequent, result in complete interruption of the adaptive workflow and thus warrant distinct attention in both analysis and reporting. Following the analysis of aborted sessions, remaining FMs were categorized into system-driven, patient-driven, and treatment execution–related groups. This framework was developed to organize and interpret the diverse types of issues encountered during oART delivery, with the goal of identifying patterns and informing targeted improvements.

## 3. Results

Table 1 lists the type, prescription, and distribution of pelvic oART cases analyzed in the study. Through a collaborative and systematic review of over 1000 pelvic oART sessions, we identified FMs and irregularities across the adaptive workflow. The multidisciplinary approach facilitated a comprehensive evaluation of these FMs and irregularities, highlighting their potential impact on patient outcomes and informing targeted risk mitigation strategies. This continuous feedback loop not only addressed existing workflow vulnerabilities but also enhanced the overall robustness and safety of the oART process, driving improvements in treatment quality and reliability.

These oART sessions typically lasted between 20 and 40 min from start to finish. Over time, we observed a trend toward improved efficiency, with session durations gradually decreasing as experience with the workflow increased and processes were refined from earlier years to more recent ones. Although the workflow differed from conventional radiotherapy and was new to our team, over 99% of adaptive sessions were delivered successfully. All patients completed their prescribed treatment. Dosimetric and clinical data will be reported elsewhere. A small number of sessions (nine sessions) were aborted and re-attempted due to identified FMs, while irregularities were noted and addressed in some other sessions.

Our study identified a range of FMs and workflow irregularities in CBCT-guided oART, spanning both documented challenges and novel issues. While some FMs align with those previously reported in the literature, others arise from the unique combination of automation and system constraints required in CBCT-based oART. The root causes of these FMs are multifaceted, primarily stemming from software-driven processes, workflow automation, patient-specific anatomical variations, and interactions across multiple treatment steps.

Section 3.1 will first describe treatment session terminations caused by severe FMs or irregularities that prevented plan execution. Section 3.2 will then explore the remaining FMs, their clinical implications, and strategies for mitigation.

### 3.1. Aborted Sessions

In this section, the FMs that led to session abortion are described. Table 2 lists these FMs as well as their occurrence frequencies before and after intervention, with details described in the following paragraphs.

**Figure 2:**
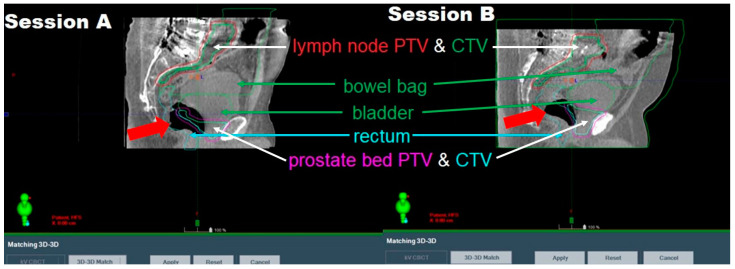
Two sample aborted sessions (Session A and Session B) due to large intrafractional gas changes for two different patients receiving prostate bed oART. Shown are the post-planning CBCT acquired immediately prior to treatment delivery superimposed with the on-couch contours delineated based on the pre-planning CBCT at the start of the oART session. The color-coded legends point to the oART contours and not the shown anatomy. In both cases, big gas bubbles (indicated by the big red arrows) substantially deformed the prostate bed targets and OARs like rectum and bladder, leading to session abortion.

**Severe intrafractional rectal gas change.** The most common cause of an aborted session in our series was the large intrafractional gas movement. These types of changes are detected on the post-planning CBCT taken immediately before the treatment. When such gas changes are small and negligible, treatment delivery is usually continued after applying the optimal alignment shifts. However, when such changes are severe and substantially deform the targets and/or organs at risk (OARs), the treatment is aborted and reattempted after the patient tries to pass the gas. This occurred for four sessions in our series. Figure 2 depicts two examples of such aborted sessions. To address this and other gas-related FMs, patients are reminded of our standard diet instructions for prostate patients, given at the initial consult, on reducing gas-producing foods to reduce rectal gas formation. In cases where diet modifications are insufficient, patients are prescribed appropriate medications such as simethicone (Gas-X) to alleviate gas buildup. For patients presenting with persistent gas during treatment sessions, real-time rectal gas extraction is considered as a last resort to ensure anatomical consistency and optimal treatment accuracy.

**Patient unable to hold the bladder.** Two sessions were aborted due to patients being unable to hold their bladder, requiring them to be brought off the treatment table and the sessions restarted after releasing and refilling the bladder. To address this FM, patients are provided clear instructions on the timing and amount of water intake prior to treatment sessions. At the simulation, an effort is also made to ensure the patient bladder is comfortably full for the oART patients (slightly different than for our standard IGRT pelvic patients, as the treatment sessions are typically shorter for those patients) rather than excessively distended, especially for patients with bladder-related challenges or symptoms to simulate with a moderately full bladder rather than a full bladder.

**Figure 3:**
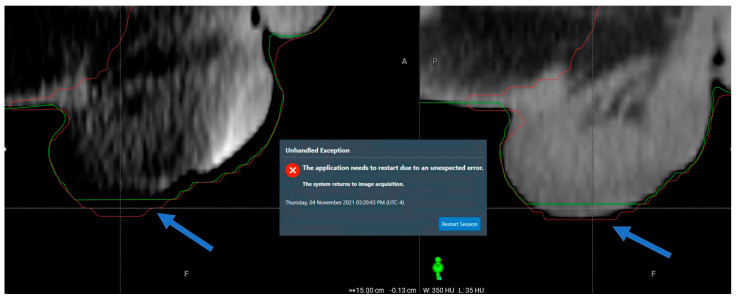
One example case where the system crashed when it failed the consistency check following manual target editing, because an automatically propagated non-target superficial structure extended beyond the body. The blue arrows point to the parts with inconsistent structures.

**Process termination from contour extending beyond the body.** Two sessions were aborted due to software crash when superficial contours extended beyond the external body contour. The crash happens when the system performs a consistency check after contours are manually edited. This consistency check is not performed if the automated contours are accepted without modification. Figure 3 shows an example instance where the body contour erroneously fell short of the patient’s scrotum contour. The other similar instance involved a patient’s colostomy bag that was placed at a different position on-couch than at the time of the simulation. These events occurred early in our experience using Ethos v1.0. Since then, we have introduced a planning step on cropping superficial structures near the body surface, which has successfully prevented recurrence.

**Figure 4:**
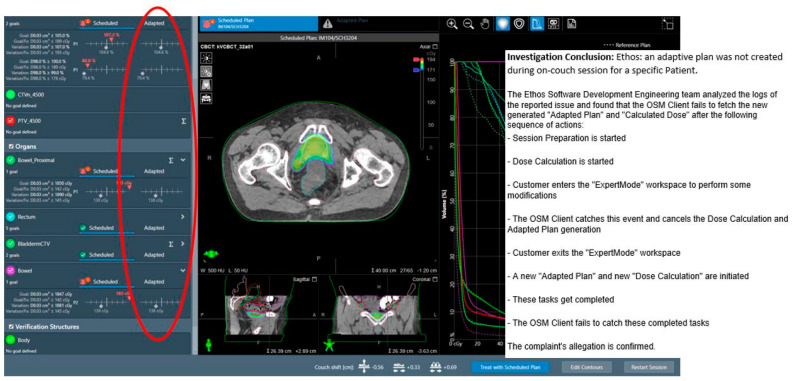
A screenshot of the single instance where the adaptive plan was unavailable after the planning process due to internal software communication issues. The session was treated using the scheduled plan. The red elliptical highlights the unavailable adaptive plan.

**Adaptive plan unavailability due to internal software communication issues.** There was one session not technically aborted, but the scheduled plan was treated due to the unavailability of the adaptive plan after about 20 min oART imaging and planning workflow. Follow-up investigations by the vendor revealed that abnormal internal software communication issues caused it, namely the system failed to catch the completion of adaptive plan generation and dose calculation and proceed to the subsequent steps. Figure 4 shows a screenshot of that session and relevant vendor investigation results. This was a rare instance that occurred with Ethos v1.1, and the vendor stated that no other occurrence like this had been reported.

### 3.2. Other FMs and Irregularities

Beyond treatment session terminations discussed in Section 3.1, additional FMs and workflow irregularities were identified in CBCT-guided oART. Some of these FMs align with challenges reported in prior adaptive radiotherapy studies for platforms using other imaging modalities, while others are unique to the CBCT-based system and its automated workflow. Unlike conventional IGRT, where manual intervention allows for more user control, the structured automation in oART can introduce unintended consequences, such as rigid target localization and deviations in fallback plans. These system-driven constraints, combined with patient-specific anatomical variations, create new complexities that require careful evaluation.

To systematically assess these challenges, we categorize the identified FMs into three groups: (1) system-driven issues, which include workflow automation constraints, software-driven errors, and planning system limitations; (2) patient-driven challenges, which arise from interfractional and intrafractional anatomical variations affecting plan adaptation; and (3) treatment planning and execution failures, which impact plan selection, dose accuracy, and the effectiveness of fallback strategies. Within each category, both previously reported and newly identified FMs are discussed, with special emphasis on novel findings that have not been documented in the literature. Understanding these FMs is essential for refining CBCT-guided oART workflows, improving clinical decision-making, and ensuring treatment safety and efficacy. While these FMs are grouped based on their primary contributing factors, it is important to note that some FMs arise from a combination of factors across multiple categories. For clarity and to avoid redundancy, each FM is described in the most relevant section based on its predominant cause, even if contributing factors span multiple domains. The following sections provide a detailed discussion of these FMs, their clinical implications, and potential mitigation strategies. Table 3 lists these FMs and their occurrence frequencies before and after intervention, with details discussed in the following paragraphs.

#### 3.2.1. System-Driven Issues

**Failure of automated Boolean structure propagation in oART planning.** Derived Boolean structures using the “auto margin combination” tool play a crucial role in oART treatment planning, serving as essential optimization structures. For example, “bladder minus CTV” is commonly used in place of the full bladder in our pelvic planning to prioritize target coverage while maintaining OAR sparing. However, certain operator errors can lead to FMs where these structures fail to propagate correctly, disrupting the adaptive workflow.

One common FM occurs when a derived Boolean structure is manually edited, which inactivates the automated derivation instructions. As a result, the structure is no longer automatically propagated during oART. To address this, our workflow includes verification steps during reference planning to ensure structures remain correctly derived. In the Ethos system, properly derived structures are indicated by a check mark next to the sigma symbol and are listed as “arithmetically calculated” in the RT Intent report.

A second, more subtle FM arises when a planner incorrectly uses a manual Boolean operation (via the “static” margin/combination button) instead of the correct “auto” tool. While manual Boolean operations are intended for fixed calculations, such as summing two kidneys into a single structure, derived Boolean structures must be instruction-driven for oART automation. Although both operations share the sigma symbol, they are located in different areas of the system interface, making a user unfamiliar with the system prone to this error and the error difficult to detect visually. A manually created structure may appear correct in the reference plan but will not regenerate during oART. Unlike improperly edited derived structures, these lack any clear visual warning, making them harder to detect.

To mitigate both errors, a new workflow step requires checking for the “arithmetically calculated” descriptor in the RT Intent report to confirm the structure was generated using the correct automated tool. These FMs, while specific to the Ethos platform, highlight broader challenges in automation transparency. They underscore the need for intuitive software design with clearly differentiated tools for manual and automated operations to reduce user error.

By incorporating these verification steps into clinical practice, we enhance the reliability of derived structures and support seamless execution of oART plans.

**Figure 5:**
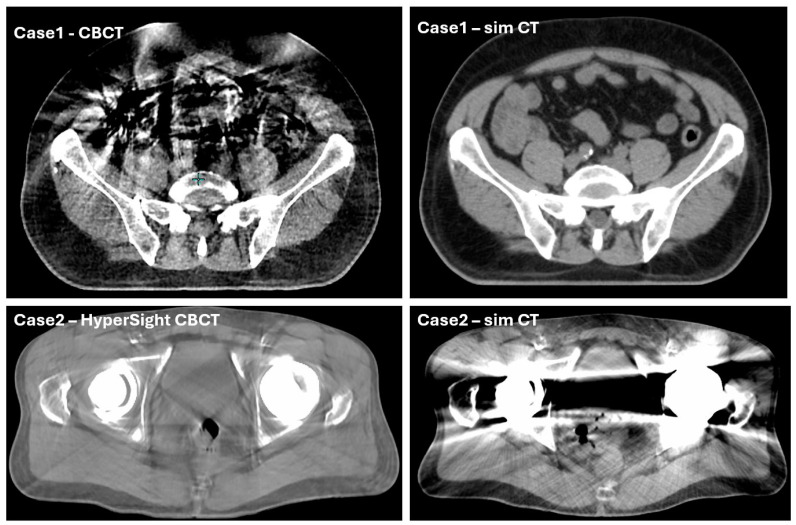
Two example cases. The upper panels show a case with considerable imaging artifacts on Ethos CBCT from abdominal gas (left) compared with the corresponding simulation CT image (right). Such imaging artifacts can affect the efficiency and accuracy of influencer and target/OAR contouring for oART. The lower panels show another case where Ethos HyperSight CBCT provided good image quality on a patient with bilateral hip implants (left), compared with the corresponding simulation CT image (right). The improved image quality and artifact reduction more effectively support the oART workflow.

**Reduced auto-contouring accuracy due to imaging artifacts.** In the pelvic region, CBCT artifacts are common with metallic implants and due to abdominal air pockets coupled with respiratory motion. The autogenerated influencer and target contours tend to be less accurate when such artifacts are severe and close to these structures, necessitating more involved inspection and manual editing steps. For example, auto-segmented bowel structure has exhibited a strong sensitivity to the specific imaging parameters utilized and to artifacts present within the pre-planning CBCT. Namely, motion artifacts coupled with pockets of bowel gas and pre-planning CBCT longitudinal extent combined with iterative CBCT or metal artifact reduction reconstruction techniques have caused large variations in the quality of the AI-generated bowel structure. This increases the on-couch session duration and potentially decreases the contour accuracy. To mitigate this, CBCT acquisition modes, such as with or without iterative reconstruction, and the technique parameters are optimized for individual cases. More effectively, the upgrade to HyperSight CBCT substantially improved the image quality and reduced imaging artifacts. The vendor has already released improved AI models for auto-segmentation of bowel loops. With the release of Ethos v2.0, further improvements in these models could be realized. Figure 5 shows one example image on a patient with considerable abdominal gas acquired with the original Ethos CBCT (upper left panel), and one example image on a patient with a bilateral hip implant acquired with the HyperSight CBCT (lower left panel). The corresponding simulation CT images, acquired with a GE LightSpeed-RT16 scanner (GE Healthcare, Boston, MA, USA), are also shown on the side to provide an image quality reference.

**Inaccurate air mapping from CBCT to sCT.** Ethos sCT generally demonstrates reasonable anatomical accuracy when compared with the input CBCT. However, one notable exception is air mapping, where considerably larger discrepancies were observed between the sCT and the CBCT. This irregularity could stem from the limitations of the deformable registration algorithm used for sCT generation in handling large deformations, violations of mass conservation, and a lack of texture in the air pockets. This issue is particularly relevant in pelvic oART, as air volume changes between simulation and on-couch imaging in the abdominal and pelvic regions are common. Such changes may occasionally occur near or overlap with the treatment targets. Our earlier study examined the dosimetric impact of these mapping errors and concluded that, in most cases, the effects were negligible [26]. Strategies to mitigate these discrepancies have been discussed earlier in the “Severe intrafractional rectal gas change” section.

**Figure 6:**
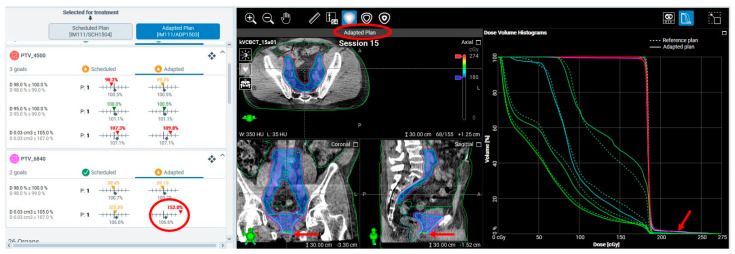
An example session in which the adaptive plan with an extreme hotspot dose at 152% of prescription was created because the MLC ran out of its maximum longitudinal range to cover the inferior extent of the PTV. The red circles and arrows highlight the portions of extreme hotspots and doses.

**Extreme adaptive plan hotspots due to limited maximum MLC field size.** In two sessions for a patient receiving prostate bed and lymph node oART, adaptive plans exhibited extreme hotspots exceeding 150% of the prescription dose at the inferior end of the prostate bed PTV (see Figure 6 for an example). These hotspots rendered the adaptive plans unsuitable for treatment, especially given that the intended hotspot dose constraint was 107%. Further investigation revealed a routine setup shift of 1.5–2 cm superiorly in these sessions, which caused the PTV extent to exceed the aperture FOV of the MLC at the inferior end of the prostate bed target.

The Ethos machine has a maximum field length of 28 cm, typically sufficient for pelvic radiotherapy with lymph nodes. For the scheduled plan, the system automatically calculates a shift to align the daily and reference PTVs. In adaptive planning, however, it assumes reoptimization will adjust for any positional differences. In situations where the target is sufficiently covered by a single beam, but an unexpectedly large longitudinal shift is required, the adaptive plan may not be able to achieve the planning directive goals without shifting the patient to sufficiently cover the intended target. This suboptimal placement limits the effective MLC span, particularly when combined with a large superior patient setup shift.

While most patients usually exhibit smaller setup shifts, systematic shifts of such an amplitude are not entirely out of the expected range for pelvic patients. To address this FM, an anticipatory 1.5 cm superior patient shift was applied when positioning the patient on the table. This adjustment reduced the likelihood of large on-couch superior shifts during the oART workflow. Following this systematic intervention, the issue did not recur in this patient.

As a general solution, the adaptive team remains vigilant about the MLC field length and target longitudinal span. In cases with minimal leeway, customized beam geometries imported via third-party planning systems (such as Eclipse used in our workaround workflow) are used to optimize isocenter placement at the center of the field or to force the treatment plan to split the plan into an auto-feathered, two-isocenter geometry. These strategies mitigate similar occurrences and enhance adaptive planning reliability.

**Figure 7:**
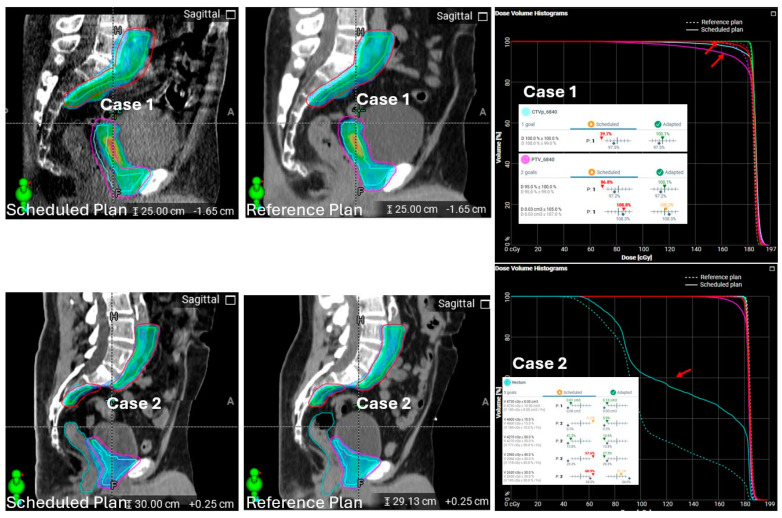
Two example cases illustrating how streamlined rigid target alignment caused substantial dose discrepancies in scheduled plans. Each case includes sagittal views of the scheduled and reference plan dose distributions (colorwash for doses ≥ prescription) and DVHs of relevant structures. Case 1 (upper panels): Highlighted by the red arrows on the DVHs, rigid alignment resulted in substantial underdosing of prostate bed targets (CTV_6840, cyan; PTV_6840, magenta). Case 2 (lower panels): Alignment, combined with organ deformation, led to rectal overdose (rectum contour, light blue), highlighting the impact of automated matching constraints on scheduled plans.

**Streamlined alignment process causing large dose-coverage or dose-sparing discrepancies in the scheduled plan.** Scheduled plans are used as the “fallback” plans in oART, based on the intuitive expectation that they will perform similarly to non-adaptive, conventional IGRT plans. However, irregularities were observed in the scheduled plans for some oART patients. Figure 7 shows two example cases, where the scheduled plan considerably underdosed the posterior portion of the prostate bed target in Case 1 and overdosed the rectum in Case 2 as well as underdosing the anterior portion of the prostate bed target, compared with the reference plans.

RCA identified the cause of this FM as the hardcoded auto-matching in the oART workflow, where the target localization does not allow the user’s manual controls as in conventional IGRT. Instead, the target localization is hardcoded based on maximizing the percentage overlap of the total target volumes from the anatomy of the day to the reference targets. There is no ability to place a priority on the target volumes of interest (e.g., prioritize match to the fossa over the nodes). In these two example cases, there was a large flex of the anatomy leading to non-rigid relative positions of the prostate bed target vs. the lymph node target. The lymph node target was larger than the prostate bed, which skewed the system’s hardcoded alignment. As a result, anterior–posterior misalignment caused dose discrepancies affecting both the target and the rectum. This FM is in contrast to conventional IGRT, where therapists would have the flexibility of aligning to a selected target (the prostate bed target) or manually aligning to the prostate bed/rectal border as is the standard practice in our clinic. Therapists would also be able to more easily visualize and detect large posture issues and reset up the patient if necessary than in the oART workflow. The oART workflow does not allow for an overlay visualization of the daily imaging compared to the reference imaging. The only indicator to the clinical team of a sub-optimal misalignment is the dose overlay and DVH. This change in how the match information is conveyed to the end users may be easily missed if the default assumption is that the scheduled plan is effectively the same workflow as standard IGRT.

The adaptive plans for these types of cases are not vulnerable to this FM, as a new plan is generated based on the session targets. However, as there are many reasons, some discussed in this work, that the adaptive plan may not be suitable or available for the session treatment, the scheduled plans need to fulfill the expectation that they are as good as conventional IGRT deliveries to offer a robust fall-off.

#### 3.2.2. Patient-Driven Challenges

**Inadequate on-couch anatomy imaged, leading to workflow interruption.** The Ethos system allows extended CBCT, providing a sufficient longitudinal field of view (FOV) to cover the entire treatment anatomy for on-couch imaging. In practice, the longitudinal CBCT range is often minimized to reduce imaging dose and acquisition time. On-couch sessions have been restarted when insufficient on-couch anatomy was imaged, particularly in cases with unfavorable daily longitudinal shifts. In these cases, the oART process could not proceed to adaptive plan generation after the CTV and other structures had been reviewed and edited due to insufficient imaging to generate the PTV. To address this FM, slightly more generous FOV is used for on-couch CBCT acquisition, especially for patients who tend to have larger daily superior–inferior shifts.

**Intrafractional anatomy changes affecting treatment dose.** Due to the extended duration of the oART workflow, which is often longer than that of conventional IGRT, patients may experience more intrafractional anatomical changes, primarily from organ filling and gas fluctuations in the pelvic region. While severe gas-related changes, as previously discussed, can lead to treatment abortions, other anatomical shifts may still alter the delivered dose, potentially offsetting or negating some of the dosimetric benefits of oART.

**Figure 8:**
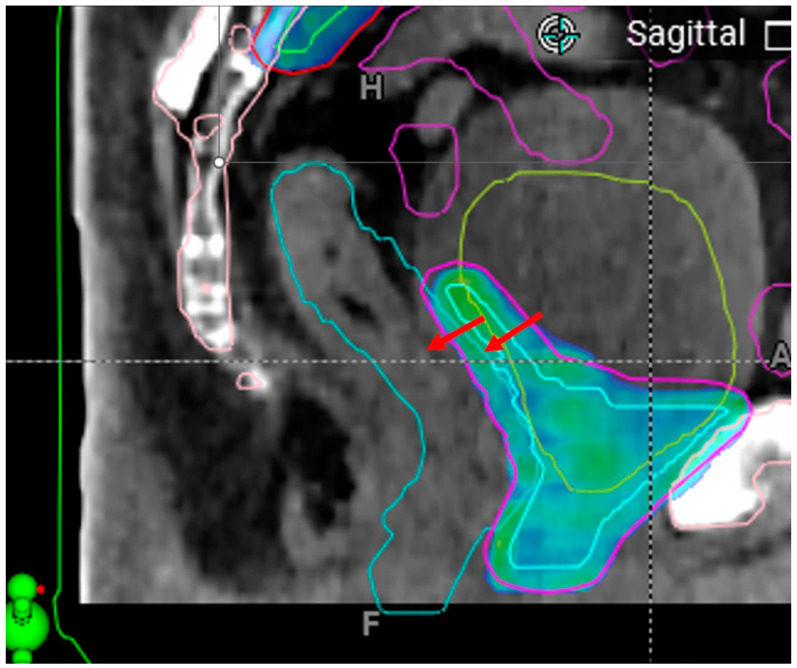
Illustration of an example case where post-planning CBCT revealed a substantial increase in bladder volume, along with deformation of the rectum (light blue), bladder (grass green), bowel (magenta), and prostate bed targets (CTV, cyan, and PTV, magenta) compared to the pre-planning CBCT contours used for oART planning. The image shows the post-planning CBCT, while contours and dose distribution (colorwash for doses ≥ prescription) correspond to the adaptive plan based on pre-planning CBCT. Arrows highlight the intrafractional anterior rectal border change.

Figure 8 illustrates a case where post-planning CBCT revealed a substantial increase in bladder volume, along with deformation of the rectum, bladder, bowel, and prostate bed target compared to the pre-planning CBCT contours used for oART planning. This variability is not an irregularity but rather an inherent limitation of oART without real-time adaptation. These intrafractional changes are patient- and session-dependent, with prolonged session durations further increasing bladder volume and the likelihood of organ deformation. Additionally, some advanced image acquisition and reconstruction modes, such as extended range and metal artifact reduction, could add additional time to the oART process, necessitating a tradeoff. In our practice, the use of such advanced modes is often reserved for the pre-planning CBCT, while the faster, basic modes are often used for the post-planning CBCT.

To mitigate these challenges, patient education and communication strategies are employed to promote standardized water intake and dietary routines. Additionally, treatment appointment times may be optimized to help patients maintain a more stable anatomy, such as avoiding early morning. Efforts to streamline the oART workflow focus on minimizing session duration by optimizing the steps for the best quality/time tradeoff and reducing wait times for involved personnel.

#### 3.2.3. Treatment Planning and Execution Failures

**Manual contour selection mismatch.** In our clinical workflow, contours are not delineated in the Ethos treatment planning system but imported from a third-party software more familiar to our physicians and planners, Eclipse (Varian Medical Systems, Palo Alto, CA, USA). This practice is fairly common due to factors such as convenience, familiarity, software capabilities, and the availability of AI support. However, since contour matching during import is a manual process, operator errors can introduce mismatches. This failure mode is critical, as it affects all subsequent workflow steps, yet it can be difficult to detect, making robust verification essential. To address this, we include in our workflow a checklist item for both the planner and the physicist checking the plan to verify contour correctness and accuracy.

**On-couch optimization structure generation.** During the reference plan creation process, it is sometimes necessary to use optimization structures generated via expansions and Boolean operations using the radiation target and surrounding OARs. An example of such a structure that our workflow has used is one that denotes the proximal sigmoid, which is generated via taking the overlap of the sigmoid with an expansion of the prostate bed clinical target volume. The intent of a structure such as this is to generate based on the daily variation of both the sigmoid as well as the bladder. On days where the patient has an emptier bladder, more of the sigmoid can move closer to the prostate bed target volume, and this structure will aid in the re-optimization process to protect this sensitive organ. The problem can arise if our structure generation rule is not sufficiently robust. Poorly defined optimization structures can create local hotspots during re-optimization. These hotspots may exceed clinical acceptability. To address this issue, more robust rules to prevent this from occurring have been developed and implemented.

**Monitor unit (MU) deviations in adaptive plan compared to reference plans.** MUs of Ethos adaptive plans can sometimes be quite different from those of the reference plan. In about 10–15% of our sessions, the adaptive plans had MUs >20% different from those of the corresponding reference plans, and often there were no observed matching target volume or intuitive OAR changes. This irregularity raised questions on the validity of the adaptive plan. Our earlier study investigated this MU variability, the results of which suggested that the variability seemed to stem from stochastic variations in the Ethos planning engine rather than from correlations with changing anatomy, but the adaptive plans were still valid with accurate dose calculation [27].

**Figure 9:**
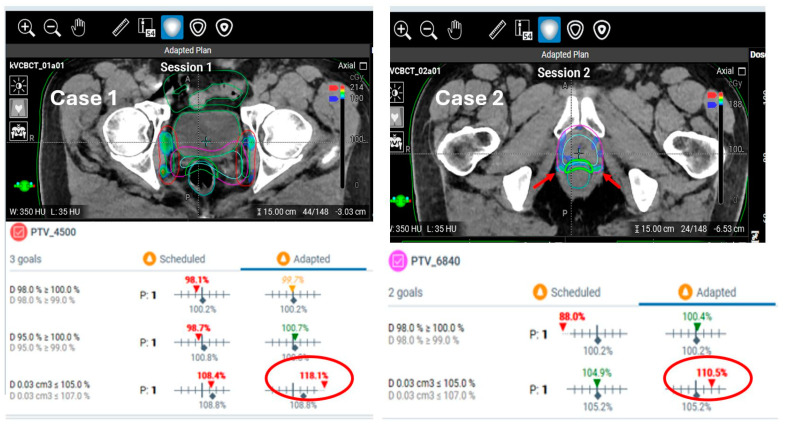
Two example cases where the adaptive plans had unacceptable target hotspots due to magnitude (Case 1) or location (Case 2). For each case, an axial view is shown for the adaptive plan displaying the hotspot dose in colorwash and relevant contours. In Case 1, the adaptive plan was rejected by the radiation oncologist due to a PTV_4500 hotspot dose of 118.1%. In Case 2, the adaptive plan was rejected due to the location (proximity to the rectum) of the hotspots with otherwise borderline acceptable magnitude of 110.5%. The red arrows highlight the locations of the hotspots, and the red circles highlight the magnitudes.

**Unacceptable hotspots in adaptive plans due to planning system variability.** Ethos adaptive plans generally result in improved target coverage and better sparing of OARs compared to the scheduled plan. However, one common reason physicians may opt not to use an adaptive plan is the presence of unacceptable hotspots. For our pelvic oART cases, a hotspot of less than 110% is required, with less than 105% being the preferred goal. However, in some instances, adaptive plans have been generated with hotspots ranging from 110% to 115%, and occasionally even close to 120%. Additionally, even when the hotspots are within acceptable thresholds (<110%), their location in unfavorable regions, such as near critical structures, can lead to the adaptive plan being rejected. Figure 9 provides example cases where the physician chose to treat with the scheduled plans despite the adaptive plans offering better target coverage and OAR sparing. These irregularities are likely due to the difficulty of using a single set of optimization constraints or planning directives to consistently address all daily anatomical variations. To address this issue, oART reference plans are occasionally revised with updated planning directives that are better suited to the observed anatomical changes during the treatment course.

**Limitations of QA software logfile analysis in handling treatment delivery interruptions.** In our workflow, post-treatment logfile QA is performed using Mobius (Varian Medical Systems, Palo Alto, CA, USA) after each adaptive plan delivery. However, the software is unable to properly process treatment sessions that experience delivery interruptions such as machine software or hardware interlocks, as it cannot merge multiple log files or distinguish between complete and partial records. As a result, Mobius QA incorrectly flags these cases as failed due to incomplete data. Currently, the vendor workflow lacks a built-in solution for this issue. In addition to generating false failures, this limitation can occasionally cause Mobius to freeze, requiring a system reboot before it can be used for subsequent patients and sessions. Addressing this limitation is essential to ensure accurate QA assessments and maintain workflow efficiency in CBCT-guided oART.

## 4. Discussion

The integration of FM and RCA is essential for a new radiotherapy system and an unconventional workflow like CBCT-guided oART. The shift from conventional IGRT to a more automated and streamlined adaptive system introduces new procedural elements that require careful oversight. While automation enhances efficiency and streamlining, it also presents unique challenges, necessitating a structured approach to error detection and mitigation. Our study demonstrated that lessons learned from a comprehensive review of clinical practice are invaluable in identifying and addressing these challenges. A multidisciplinary approach involving radiation oncologists, medical physicists, medical dosimetrists, and radiation therapists is instrumental in fostering continuous improvement and ensuring safe implementation.

Several recent studies have explored risks and workflow considerations in CBCT-guided oART, primarily through prospective risk analyses, FMEA, and checklist-based safety protocols [21,22,23,24,25]. While these efforts have identified key challenges such as cognitive biases in decision-making, limitations in system interoperability, and the impact of automation on treatment planning, they often rely on theoretical assessments or early practice experience. Our study advances this field by providing a comprehensive practice-based evaluation of FMs based on over 1000 pelvic oART fractions, offering a structured, retrospective analysis that captures both anticipated and previously undocumented workflow irregularities. Unlike prior work that focused mainly on pre-treatment risks, we categorized FMs into system-, patient-, and execution-related types. This approach highlights how closed-loop automation can restrict workflows and affect accuracy and efficiency.

Certain FMs, such as severe intrafractional gas changes, are discussed in our paper in more than one context to reflect their variable clinical consequences. For example, when gas change led to session abortion, it is detailed within the ‘Aborted Sessions’ section. However, when similar changes were managed intra-session and did not result in abortion, they are discussed within ‘Patient-Driven’ FMs due to their impact on dose accuracy. Our approach was to present these FMs within the most contextually appropriate category while noting their broader relevance across multiple stages of the workflow. This also underscores the importance of nuanced understanding and management strategies for recurring yet variably impactful FMs.

One of the key findings of our study is that the closed-loop automation in the CBCT-guided oART system introduces unexpected workflow irregularities and FMs. While automation aims to reduce human intervention and variability, certain system constraints, such as rigid alignment protocols and software limitations, have led to unintended consequences. Some failures result from software issues like unexpected image registration or alignment errors. These are difficult to predict during simulation. Understanding these novel FMs and incorporating corrective strategies into routine clinical practice are crucial for optimizing the workflow and maintaining treatment accuracy.

Another major consideration is the robustness of fallback plans. While scheduled (non-adaptive) plans are intended to serve as a safety net when the adaptive plans are not viable or suitable, our findings highlight that scheduled plans may not always perform equivalently to conventional IGRT plans. The streamlined alignment process in oART may lead to significant dose discrepancies in scheduled plans, particularly when complex anatomical changes occur. Ensuring that fallback plans maintain treatment quality is critical, necessitating alternative patient setup approaches when required. Furthermore, the impact of intrafractional anatomical changes, which are more pronounced in longer oART workflows, must be acknowledged and incorporated into mitigation strategies.

Although some of the FMs described in this paper are specific to the Ethos platform, they illustrate nuanced and high-impact failure modes that are difficult to anticipate through prospective FMEA alone. Understanding these subtleties is critical to enhancing system robustness, ensuring workflow safety, and guiding improvements across oART platforms.

Our findings add to the growing body of literature on oART by providing a comprehensive practice-based evaluation of system-specific FMs. While several prior studies largely focused on theoretical or prospective assessments, our clinical practice experience underscores the importance of retrospective analysis to identify unforeseen challenges in daily practice. The insights gained from our study contribute to the ongoing refinement of CBCT-guided oART workflows and offer guidance for future technological advancements in adaptive radiotherapy platforms. The lessons learned from this work are expected to inform the next generation of adaptive radiotherapy systems and workflows, further improving automation, robustness, and clinical efficacy.

## 5. Conclusions

CBCT-guided oART represents a transformative approach in radiation therapy, enhancing treatment delivery precision and accuracy considering changing patient anatomy. In our review of over 1000 pelvic oART sessions, we identified specific FMs, including session-aborting events and more frequent system-, patient-, and workflow-driven issues, that impact treatment accuracy, efficiency, and safety. These FMs were analyzed to understand their causes and to recommend targeted corrective or preventative measures. While some FMs were platform-specific, many reflect broader challenges relevant to any oART system. These findings underscore the importance of robust system design, effective fallback mechanisms, and continuous QA. A multidisciplinary approach and iterative refinement of workflows are essential to safe and effective implementation. Our experience provides practical insights for clinicians, future adopters, and vendors aiming to improve the safety and accuracy of oART.

## Figures and Tables

**Table 1:** Pelvic oART cases treated.

Type	Dose and Fractionation	Number of Cases (% of Total Cases)
Prostate bed, conventional	1.8 Gy * 38 fx (25 fx, phase 1 to prostate bed + LN^%^ & 13 fx, phase 2 to prostate bed)	30 (60%)
Pelvic SBRT (LN, intact prostate, prostate bed)	5, 7, or 7.25 Gy * 5 fx	9 (18%)
Intact prostate, conventional #	1.8 or 2 Gy * 20, 28, or 39 fx	5 (10%)
Other pelvis (bladder, rectum, etc.)	1.8, 2, or 2.75 Gy * 20, 27, or 30 fx	6 (12%)

^%^LN: lymph nodes, # Some of these patients also received a high dose rate brachytherapy boost.

**Table 2:** Ethos oART FMs that led to session abortion in our series.

FM	Frequency Before Intervention/Prevention	Frequency After Intervention/Prevention
Severe intrafractional rectal gas change	1% (5 instances)	0.6% (2 instances)
Patient unable to hold the bladder	0.3% (2 instances)	0% (0 instance)
Process termination from contour extending beyond the body	1% (2 instances)	0% (0 instance)
Adaptive plan unavailability due to internal software communication issues	0.1% (1 instance)	N/A

**Table 3:** Other Ethos oART FMs in our series.

FM Category	FM	Frequency Before Intervention/Prevention	Frequency After Intervention/Prevention
System-driven	Failure of automated Boolean structure propagation in oART planning	10% (3 plans)	0%
	Reduced auto-contouring accuracy due to imaging artifacts	20% estimated with varying severity, not quantified	<5% estimated, not quantified
	Inaccurate air mapping from CBCT to sCT	20% estimated with varying severity, not quantified	10% estimated, not quantified
	Extreme adaptive plan hotspots due to limited maximum MLC field size	Two instances *	Never occurred after intervention
	Streamlined alignment process causing large dose-coverage or dose-sparing discrepancies on the scheduled plan	4%	NA
Patient-driven	Inadequate on-couch anatomy imaged, leading to workflow interruption	0.2%	0%
	Intrafractional anatomy changes affecting treatment dose	40% estimated with varying severity, not quantified	30% estimated, not quantified
Treatment planning and execution failures	Manual contour selection mismatch	1 instance caught at checking	0
	On-couch optimization structure generation	3%	1%
	Monitor unit (MU) deviations in adaptive plan compared to reference plans	16% with >10% MU deviation in v1.0 and 1.1	Stopped using it as a metric for ART plan assessment
	Unacceptable hotspots in adaptive plans due to planning system variability	10% estimated, not quantified	5% estimated, not quantified
	Limitations of QA software logfile analysis in handling treatment delivery interruptions	0.2%	NA

* Both instances occurred in a single patient during fractions 15 and 16. The rest of this patient’s treatments were free of this issue after implementing the intervention from fraction 17.

## Data Availability

Data are contained within the article.
